# Recto-Vesical Lithotomy

**Published:** 1831-01-01

**Authors:** 


### RECTO-VESICAL LITHOTOMY.

OUR readers may be aware that this operation which was first performed by

Sanson in France, and did not experience a very cordial reception or extensive patronage in its native country, has latterly become the vogue in Italy. It has also been tried on one or two occasions in this country, but so many plausible objections have been urged against it, that it is not likely to be commonly adopted. In the Transactions of the Medical and Physical Society of Calcutta, a case is related by Dr. Waddell, in which a stone was extracted from the bladder by this operation.

*Case.* A boy, about nine years of age, born of Mussulman parents settled in the town of Rangoon, had been affected with the ordinary symptoms of stone in the bladder for seven years. On examination a stone was discovered by the sound, and by the anus. The recto-vesical operation was proposed and performed on the following day, by Mr. Tweedie, field-surgeon to the Bengal division of the army.

The boy being placed near the end of a high table, in the usual position for lithotomy, and the thighs being held apart by assistants, the operator introduced the fore and middle fingers of his left hand into the rectum so as to embrace and fix the stone, and with a scalpel, having its cutting edge defended with cloth wrapped round it till within half an inch of its extremity, an incision about $\frac{3}{4}$ of an inch long was made into the shoulder and neck of the bladder, in the direction of the raphe; the scalpel being then withdrawn, the stone was brought away by a pair of common ball forceps. Several impediments occurred during the operation, such as the evacuation of the patient's bladder and bowels, the bearing down of the transverse folds of the rectum, and the recession of the calculus before the forceps; nevertheless the time occupied did not exceed five minutes, and not above half an ounce of blood was lost. As soon as the little fellow was released from the table, he had another stool, with some degree of straining; but all uneasiness quickly subsided, and before we left the house he seemed to feel as if nothing was the matter with him.

The stone was of a conoid form, four and a half inches in length, three and two-tenths of an inch in circumference at its greatest diameter, and weighed six drachms. Its surface was rough and granular, and its appearance that of the triple phosphate. The urine after the operation became feculent and the stools urinous—on the tenth day he was out of bed and walked about the house—after this he became feverish, with pain in the umbilicus, and passed a live lumbricus, six inches long, by the urethra, when the urine was bloody for some days, and voided with straining. On the thirty-third day the urine was still mixed at times with fæces, and interrupted in its discharge by flatus At. night, when asleep, the urine was occasionally voided by the rectum. In about six weeks after the operation the communication between the rectum and bladder closed.

Dr. Waddell appends some extremely laudatory observations on the recto-vesical operation, but as his practical acquaintance with it seems to be confined to the foregoing case, we may take the commendations for what they are worth. One point we cannot pass over in silence. After enumerating what he conceives to be the common modes of death from the lateral operation, but in which the Doctor is certainly wrong, he contrasts the results with those from the recto-vesical methods. "On the contrary, (*cæteris paribus*, that is, the prostate and bladder being in a healthy state, and the restorative powers of the constitution unimpaired) there is abundant evidence to shew that the improved recto-vesical operation is attended with little or no danger to life!" This passage evinces a very imperfect acquaintance with the facts on which it professes to rely. The abundant evidence is all the other way. In the Archives Generales, for July, 1824, we learn that of 69 who have undergone the recto-vesical operation, 13 have died, or rather less than one in five—and of those who recovered there are eight with fistulous openings; but of five patients operated on in the Surgical School at Turin three died, whilst eleven cut in the lateral way recovered. Dupuytren operated after Vacca's method in six instances. He lost three patients by inflammation within the pelvis, and the other three remained with incurable fistulæ. Dupuytren was asked if he would still try the plan; he made no answer, but shook his head. Riberi saw two children cut by Sanson. One died in a few days of peritonitis, and the other was given up before Riberi's departure. Barbantini, who first introduced the operation into Italy, has acknowledged its disadvantages.

But, says Dr. Waddell, it is the improved operation, which is not to be accompanied with danger. Aye, there's the rub! Which is the improved operation? Is it Sanson's first or Sanson's last—or Vacca's—or Mr. Sleigh's—is it the high or is it the low—with the division of the sphincter, or without it? Alas! we cannot tell. Perhaps Dr. Waddell intends *his* case as an instance of the improved operation. If so, we can assure him that the proceeding adopted has been denounced by all, except its inventor, if such he can be called, Mr. Sleigh. A large stone could not, and a small one should not be extracted by it. In this operation there is risk of wounding the vesiculæ—there is risk of wounding the cul-de-sac of the peritoneum—there is risk of recto-vesical fistula—there is risk of foreign bodies getting into the bladder from the rectum, and forming the nucleus of a future stone. Besides these objections, the operation is more difficult, and in Dr. Waddell's case it was so, than the lateral operation, and the convalescence is more tedious. In fact, it is agreed on all hands, that the operation performed in the present case is, for ordinary cases of stone in the bladder, a bad one.

The avowed object of the recto-vesical operation has always been, the extraction of a larger stone than could readily be removed by the lateral operation. To effect this, it is a sine quâ non that the sphincter should be divided. Several plans have been adopted; but, as far as we know, the incision in all has been in the line of the median plane, in that of the raphé of the perineum. It is a question whether by such an incision we obtain more space than we should by the lateral operation. In this, the rectum is frequently wounded whilst cutting into the groove of the staff, or in using the bistoire cachée, and the experience of surgeons has proved that such wounds of the gut are not usually dangerous. We have seen the rectum wounded in three instances, whilst performing the lateral operation, and no materially bad consequences followed. A man at St. George's Hospital was lithotomized in the usual way. Symptoms of urinous infiltration and sloughing at the neck of the bladder ensued, and in order to allow a more ready exit to the matter, urine, and sloughs, Mr. Brodie laid open the rectum from the wound. The patient who previously seemed to be dying, recovered. These observations naturally led to what we are about to mention.

A young man, at present in the hospital, had laboured under the symptoms of stone in the bladder for twenty years. Many surgeons had examined him, and all, we believe, except one, had refused to perform the lateral operation, on account of the size of the stone. That one gentleman had *not* examined the patient by the rectum. Baron Heurteloup would not drill the stone; he thought it was too large. The calculus, when felt from the rectum, appeared to be of extreme size, and its anterior extremity was partially impacted in the prostatic part of the urethra. The high operation had been proposed, but this was considered unadvisable, for if the stone was of the magnitude imagined, the sonde-à-dard could not have been employed with facility. On mature consideration Mr. Brodie adopted a modification of the lateral and rectal operations, which may prove of great service on future occasions.

Having introduced the staff he cut upon it with the knife in the perineum in the usual way, and passing it along the groove of the staff into the bladder he divided part of the prostate. He then took Blizard's knife, passed it along the staff into the bladder, completed the section of the prostate and part of the neck of the bladder, and turning the edge of the knife towards the right, nicked the wound in the prostate on that side, by which means he acquired additional room at a small expense of division of parts. The staff had lain on the right side of the stone; it was now withdrawn. Mr. Brodie took a curved probe-pointed bistoury, with a sharp-pointed bistoury blade concealed in it, and capable of being pushed forward or retracted at pleasure. He passed this to the bottom of the wound, and introduced the fore-finger of the right hand into the rectum. Having felt with this finger the button-end of the bistoury, the sharp-pointed blade was pushed forwards, and the wall of the gut penetrated on the finger. This blade was then withdrawn —the probe-pointed bistoury had entered the rectum—and its division was completed from within outwards, by drawing out the bistoury with the probe-end resting on the fore-finger, as is done in the operation for fistula ani. The opening in the rectum at the bottom of the wound was not exactly opposite to the wound in the bladder, in order that a kind of valve might be formed, to prevent the ready transit of the contents of the bowel into the bladder. The remainder of the rectum was divided on its anterior and left lateral aspect. The forceps were introduced and the stone was readily extracted. It did not prove so large as had been anticipated by all who had, on different occasions, examined the patient. It was long, its anterior end was impacted in the prostatic part of the urethra, and from being always held in this situation, it had appeared to be fixed by its magnitude, and the finger in the rectum could never reach its posterior boundary.

The wound obtained by the operation was capable of admitting the egress of a calculus of very large size. The operation itself was simple, scientific, performed without difficulty. There was little or no bleeding at the time of its performance, and although a slight secondary hæmorrhage occurred in the afternoon, it was readily arrested by pressure on the pudic artery, and has not since returned. The event cannot yet be considered as determined, but the patient's condition at present is not unfavourable. We shall give the case more in detail at an early opportunity.

